# Non-invasive Prediction of Esophageal Varices: Is It Possible?

**DOI:** 10.4103/1319-3767.74426

**Published:** 2011

**Authors:** Konstantinos C. Thomopoulos

**Affiliations:** Department of Gastroenterology, University Hospital of Patras, Patras, Greece

Varices are a serious consequence of portal hypertension, and variceal bleeding is a severe complication occurring in up to 30% of patients with cirrhosis. Despite improvement in diagnosis and therapy, mortality from acute variceal bleeding may still reach up to 20%. Moreover, it is the second most common cause of death in cirrhotic patients.[1,2] Nonselective β-blockers given to cirrhotic patients without a history of variceal bleeding reduce the risk of first bleeding and the mortality rate, and probably, isosorbide mononitrate or band ligation may also be used for preventing the first episode of bleeding in the cases of intolerance or contraindications to β-blockers. The most reliable and accurate method to detect the presence of large esophageal varices is an upper gastrointestinal endoscopy. It is now recommended that all patients with established cirrhosis should be screened by upper gastrointestinal endoscopy for the presence of varices at the time of diagnosis. Patients with large varices should be treated with nonselective β-blockers to reduce the incidence of first variceal bleeding. Also, patients without varices or with small varices should be reendoscoped every 1–3 years.[2]

However, less than 50% of cirrhotic patients have varices at the screening endoscopy and the majority have small-sized varices, which carry a very low risk of bleeding. Also a substantial number of patients will not develop large varices during screening and therefore will undergo unnecessary endoscopies which are uncomfortable, invasive, and costly. Over the years, a great effort has been made either to introduce less invasive, alternative to standard endoscopy diagnostic methods or to restrict the performance of endoscopy in high-risk patients by using a variety of noninvasive predictors.

Video capsule endoscopy could be a minimally invasive method for detecting gastroesophageal varices. Although overall agreement between endoscopy and video capsule endoscopy in detecting and grading varices is relatively high and esophageal capsule endoscopy is well tolerated, it is still not equivalent to standard endoscopy and its cost-effectiveness compared to upper endoscopy remains to be determined.[3]

Furthermore, computed tomography (CT) scanning, although not entirely noninvasive, could be a good alternative to endoscopy as it is also able to detect other pathological findings, mainly focal lesions in the liver. However, CT scanning cannot very well distinguish small from large varices nor can it detect small varices with red signs that also carry a high-risk of bleeding.[4]

Transient elastography is a new method that measures liver stiffness, and it has been shown to correlate strongly with the level of portal hypertension which is the most important factor for the development of varices. In a previous study, liver stiffness measurement was positively correlated with the presence of esophageal varices but failed to demonstrate a correlation between liver stiffness measurement and variceal size.[5]

The factors related to the presence of varices are not well-defined, but it is known that they appear after the hepatic venous pressure gradient has increased to at least 10–12 mmHg. Because minimally invasive methods cannot replace endoscopy, many studies have tried to determine whether clinical or laboratory nonendoscopic parameters could predict the presence of large esophageal varices, and whether it is possible to identify a subgroup of cirrhotic patients with a high probability of large varices, in order to improve cost effectiveness and avoid patients’ discomfort by overusing screening endoscopy.

In this issue of the *Saudi Journal of Gastroenterology*, Cherian *et al*.[6] report three nonendoscopic predictors for the diagnosis of large esophageal varices: low platelet count, Child-Pugh class, and spleen diameter, in a prospective study, where 229 newly diagnosed patients with liver cirrhosis, without a history of variceal bleeding, were included. Patients were mainly of Child-Turcotte-Pugh class B (55.5%) and the cause of cirrhosis was alcohol consumption (42.4%) followed by B or C viral hepatitis (25.3%). Overall, 178 patients had varices (77.7%) while 81 (35.4%) had large varices. On multivariate analysis Child-Turcotte-Pugh B/C, platelet count <90,000 per μl and spleen diameter >160 mm were significant predictors for the presence of large esophageal varices.

Several laboratory and radiological parameters either alone or in combination have been correlated in previous studies with the presence of esophageal varices.[7–21] However, the variables or combination of variables reported in each study differ among studies [Table 1].

**Table 1:** Characteristics of study population and noninvasive predictors of esophageal varices in different studies

	N	Causes of cirrhosis	Alcohol (%)	Child-Pugh A/B/C	Varices/large varices (%)	Noninvasive predictors
Kim BK *et al.. Liv Int.*	318	HBV	0	78/20.1/1.9	46.2/37.1	Platelet count^2^/[monocyte fraction (%) × segmented neutrophil fraction (%)
Sarangapani *et al. Saudi J Gastroenterol. 2010*	106	ALL	58.5		72.6/41.1	Platelet count Spleen size >13.8 mm portal vein >13 mm splenic vein >11.5 mm
Agha *et al.* Dig. Dis. Sci. 2009	311	HCV	0	25.8/58.6/15.6	49.5/12.9	Platelet count/spleen diameter ratio (cutoff value of 909)
Barrera *et al. Ann Hepatol.*2009	67	ALL	26.9	46.2/38.8/15	85/49.3	Platelet count/spleen diameter ratio
Hong *et al. BMC Gastroent.*2009	146	HBV	0	25.4/50.0/24.6	74.7/28.1	Portal vein diameter and spleen width
Tarzamni *et al. World J Gastroenterol.* 2008	83	ALL	2.4		81.2/22.3	Spleen size >15.05 cm Portal hypertensive index >2.08
Burton JR *et al. J Clin Gastroenterol.*2007	505	ALL	13.9	50.9/39.6/9.5	58.6/17.8	Platelet count Child-Pugh score
Levy *et al. Clin Gastroenterol Hepatol.*2007	91	PBC			37.4	Platelet count ≤ 140,000 and/or a Mayo risk score ≥4.5
Giannini *et al. Am J Gastroenterol.*2006	218	ALL	18.8	50.9/34.4/14.7	54.1/21.6	Platelet count/spleen diameter cutoff: 909
Thomopoulos *et al. Dig Liver Dis.*2003	184	ALL	42.9	64.3/27.3/8.4	50/17.9	Platelet count Spleen size Ascites
Giannini *et al.* Gut. 2003	145	ALL	24.1	37/36/27	61/20	Platelet count/spleen diameter cutoff: 909
Zaman *et al. Arch Intern Med.* 2001	300	ALL	13.3	22/58/20	67.7/31.7	PLT ≤ 90,000/ml >varices PLT ≤ 80,000/ml >large varices Child-Pugh class
Schepis *et al. Hepatology.*2001	143	ALL	10.3	59/41/0	44/19.6	PV >13 mm Prothrombin activit < 70 % Platelets < 100,000/ml
Chalasani*et al. Am J Gastroenterol.*1999	346	ALL	33	22/48/30	70/20	Platelet count < 88,000/ml splenomegaly
Pilette *et al. J Hepatol.*1999	116	ALL		50/24/26	72/44	Platelet count Prothrombin index Spider naevi

As in this study, platelet count and spleen size have been found to be associated with the presence of varices and large varices in the majority of studies. This is expected, as portal hypertension is the initial and most important factor leading to the development of varices while the presence of varices is proportional to the severity of liver disease and the degree of portal hypertension.[2] However, the results were not identical in all studies as portal vein diameter on ultrasound, the presence of teleangectasias, Child-Pugh class, prothrombin activity, ascites, and albumin levels in combination have also been implicated.

In addition, the predictive model of a combination of variables and the discriminating threshold for the presence of varices or large varices for continuous variables differ among studies. A cutoff value of 68,000–140,000 per ml has been reported for platelets. These differences may be due to different populations studied regarding the etiology of cirrhosis and/or the stage of the disease. Some studies include mainly Child-Pugh A patients while others include cirrhotic pretransplantation patients. The rate of Child-Pugh A patients varies from 22% to 78% in the cohorts included in various studies.[6–22] Also the percentage of patients with alcoholic cirrhosis in the study population may be important [Table 1]. Although in cirrhosis of viral etiology the portal pressure is relatively stable, in alcoholic cirrhosis it may fluctuate with alcohol consumption or abstinence, and, in addition alcohol consumption may have a direct effect on platelets.

Despite the adequate number of studies and the number of patients, no single parameter or combination of parameters has so far been widely established as a reliable noninvasive predictor of the presence of varices.

Furthermore, the strength of each test—the predictive model must be validated extensively in large cohorts globally before widespread use. When the predictive model provided by Schepis *et al*. was tested in an independent cohort of cirrhotic patients mainly of Child-Pugh class A, 41.6% of patients in the class with the highest probability of having varices had no varices at endoscopy whereas 34.4% of those classified in the class with the lowest probability of having varices had them.[22] Also less reliable results were obtained when Burton *et al*. assessed the previously published predictive model by Zaman and coworkers in a different cohort with less advanced liver disease.[9]

In conclusion, upper gastrointestinal endoscopy remains the gold standard for the diagnosis of gastroesophageal varices. On the other hand, it seems possible that by using noninvasive predictors we could restrict the use of endoscopy to those cirrhotic patients who are high risk for bleeding. As a diagnosis of cirrhosis is increasingly being made at a very early asymptomatic stage by noninvasive methods, this strategy is mandatory. The diagnosis of varices is not of clinical significance, *per se*, but rather the impact of any strategy on the overall prognosis of cirrhotic patients. So far, we have no data comparing, prospectively, standard endoscopy to endoscopy only in high-risk patients according to a predictive model. The impact on the prevention of bleeding and/or mortality of screening by upper gastrointestinal endoscopy only patients considered high risk for the presence of large esophageal varices must be evaluated with future prospective trials.

## References

[1] Carbonell N, Pauwels A, Serfaty L, Fourdan O, Levy VG, Poupon R (2004). Improved survival after variceal bleeding in patients with cirrhosis over the past two decades. Hepatology.

[2] Garcia-Tsao G, Sanyal AJ, Grace ND, Carey W (2007). Practice Guidelines Committee of the American Association for the Study of Liver Diseases; Practice Parameters Committee of the American College of Gastroenterology. Prevention and management of gastroesophageal varices and variceal hemorrhage in cirrhosis. Hepatology.

[3] de Franchis R, Eisen GM, Laine L, Fernandez-Urien I, Herrerias JM, Brown RD (2008). Esophageal capsule endoscopy for screening and surveillance of esophageal varices in patients with portal hypertension. Hepatology.

[4] Perri RE, Chiorean MV, Fidler JL, Fletcher JG, Talwalkar JA, Stadheim L (2008). A prospective evaluation of computerized tomographic (CT) scanning as a screening modality for esophageal varices. Hepatology.

[5] Vizzutti F, Arena U, Romanelli RG, Rega L, Foschi M, Colagrande S (2007). Liver stiffness measurement predicts severe portal hypertension in patients with HCV-related cirrhosis. Hepatology.

[6] Cherian JV, Deepak N, Ponnusamy RP, Somasundaram A, Jayanthi V (2011). Non invasive predictors of large esophageal varices. Saudi J Gastroenterol.

[7] Giannini E, Botta F, Borro P, Risso D, Romagnoli P, Fasoli A (2003). Platelet count/spleen diameter ratio: Proposal and validation of a non-invasive parameter to predict the presence of ooesophageal varices in patients with liver cirrhosis. Gut.

[8] Giannini E, Zaman A, Kreil A, Floreani A, Dulbecco P, Testa E (2006). Platelet count/spleen diameter ratio for the non invasive diagnosis of oesophageal varices. Results of a multicenter, prospective, validation study. Am J Gastroenterol.

[9] Burton JR, Liangpunsakul S, Lapidus J, Giannini E, Chalasani N, Zaman A (2007). Validation of a multivariate model predicting presence and size of varices. J Clin Gastreoenterol.

[10] Sarangapani A, Shanmugam C, Kalyanasundaram M, Rangachari B, Thangavelu P, Subbarayan JK (2010). Noninvasive prediction of large esophageal varices in chronic liver disease patients. Saudi J Gastroenterol.

[11] Chalasani N, Imperial TF, Ismail A, Sood G, Carey M, Wileox CM (1999). Predictors of large esophageal varices in patients with cirrhosis. Am J Gastroenterol.

[12] Pilette C, Oberti F, Aube C, Rousselet MC, Bedossa P, Gallois Y (1999). Non-invasive diagnosis of esophageal varices in chronic liver diseases. J Hepatol.

[13] Schepis F, Camma C, Niceforo D, Magnano A, Pallio S, Cinquegrani M (2001). Which patients with cirrhosis should undergo endoscopic screening for esophageal varices detection?. Hepatology.

[14] Tarzamni MK, Somi MH, Farhang S, Jalilvand M (2008). Portal hemodynamics as predictors of high risk esophageal varices in cirrhotic patients. World J Gastroenterol.

[15] Barrera F, Riquelme A, Soza A, Contreras A, Barrios G, Padilla O (2009). Platelet count/spleen diameter ratio for non-invasive prediction of high risk esophageal varices in cirrhotic patients. Ann Hepatol.

[16] Hong WD, Zhu QH, Huang ZM, Chen XR, Jiang ZC, Xu SH (2009). Predictors of esophageal varices in patients with HBV-related cirrhosis: A retrospective study. BMC Gastroenterol.

[17] Thomopoulos KC, Labropoulou-Karatza C, Mimidis KP, Katsakoulis EC, Iconomou G, Nikolopoulou VN (2003). Non-invasive predictors of the presence of large oesophageal varices in patients with cirrhosis. Dig Liver Dis.

[18] Levy C, Zein CO, Gomez J, Soldevila-Pico C, Firpi R, Morelli G (2007). Prevalence and predictors of esophageal varices in patients with primary biliary cirrhosis. Clin Gastroenterol Hepatol.

[19] Zaman A, Becker T, Lapidus J, Benner K (2001). Risk factors for the presence of varices in cirrhotic patients without a history of variceal hemorrhage. Arch Intern Med.

[20] Agha A, Anwar E, Bashir K, Savarino V, Giannini EG (2009). External validation of the platelet count/spleen diameter ratio for the diagnosis of esophageal varices in hepatitis C virus-related cirrhosis. Dig Dis Sci.

[21] Kim BK, Han KH, Park JY, Ahn SH, Kim JK, Paik YH (2010). Prospective validation of P2/MS noninvasive index using complete blood counts for detecting oesophageal varices in B-viral cirrhosis. Liver Int.

[22] Riggio O, Angeloni S, Nicolini G, Merkel C (2002). Endoscopic screening for esophageal varices in cirrhotic patients. Hepatology.

